# *Bartonella* infections are prevalent in rodents despite efficient immune responses

**DOI:** 10.1186/s13071-023-05918-7

**Published:** 2023-09-04

**Authors:** Ruth Rodríguez-Pastor, Adam Z. Hasik, Nadav Knossow, Enav Bar-Shira, Naama Shahar, Ricardo Gutiérrez, Luis Zaman, Shimon Harrus, Richard E. Lenski, Jeffrey E. Barrick, Hadas Hawlena

**Affiliations:** 1 Jacob Blaustein Center for Scientific Cooperation, The Jacob Blaustein Institutes for Desert Research, Ben-Gurion University of the Negev, Midreshet Ben-Gurion, Israel; 2 Present Address: Institute of Evolutionary Biology, University of Edinburgh, Edinburgh, UK; 3 The Mitrani Department of Desert Ecology, Swiss Institute for Dryland Environmental and Energy Research, The Jacob Blaustein Institutes for Desert Research, Ben-Gurion University of the Negev, 849900 Midreshet Ben-Gurion, Israel; 4 Section of Immunology, Department of Animal Sciences, Faculty of Agricultural, Nutritional and Environmental Sciences, The Hebrew University of Jerusalem, Rehovot, Israel; 5 National Reference Center for Bacteriology, Costa Rican Institute for Research and Teaching in Nutrition and Health (INCIENSA), Cartago, Costa Rica; 6 Department of Ecology and Evolutionary Biology, Center for the Study of Complex Systems (CSCS), University of Michigan, Ann Arbor, MI USA; 7 Koret School of Veterinary Medicine, Faculty of Agricultural, Nutritional and Environmental Sciences, The Hebrew University of Jerusalem, Rehovot, Israel; 8 Department of Microbiology and Molecular Genetics, Michigan State University, East Lansing, MI USA; 9 Department of Molecular Biosciences, The University of Texas at Austin, Austin, TX USA

**Keywords:** Antigen escape, Bacterial dynamics, Disease ecology, Ecoimmunology, Host–pathogen interactions, Microbial ecology, Recurrent bacteremia

## Abstract

**Background:**

Pathogens face strong selection from host immune responses, yet many host populations support pervasive pathogen populations. We investigated this puzzle in a model system of *Bartonella* and rodents from Israel’s northwestern Negev Desert. We chose to study this system because, in this region, 75–100% of rodents are infected with *Bartonella* at any given time, despite an efficient immunological response. In this region, *Bartonella* species circulate in three rodent species, and we tested the hypothesis that at least one of these hosts exhibits a waning immune response to *Bartonella*, which allows reinfections.

**Methods:**

We inoculated captive animals of all three rodent species with the same *Bartonella* strain, and we quantified the bacterial dynamics and *Bartonella*-specific immunoglobulin G antibody kinetics over a period of 139 days after the primary inoculation, and then for 60 days following reinoculation with the same strain.

**Results:**

Contrary to our hypothesis, we found a strong, long-lasting immunoglobulin G antibody response, with protective immunological memory in all three rodent species. That response prevented reinfection upon exposure of the rodents to the same *Bartonella* strain.

**Conclusions:**

This study constitutes an initial step toward understanding how the interplay between traits of *Bartonella* and their hosts influences the epidemiological dynamics of these pathogens in nature.

**Graphical Abstract:**

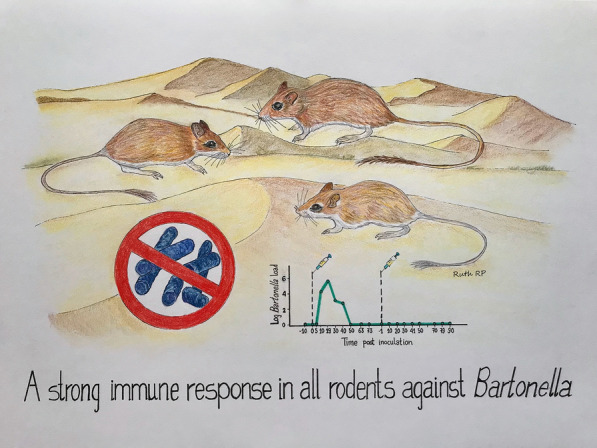

## Background

The prevalence of parasites and pathogens in their host populations is a critical ecological variable for explaining epidemiological patterns [1–3]. For example, widespread pathogens are more likely to jump to new host species, and they often spread faster than more sporadic pathogens [4]. Despite these patterns, we lack a detailed understanding of how the interplay between pathogen and host traits gives rise to the diverse epidemiological dynamics seen in nature.

The duration of infections of individual hosts is a key determinant of disease and transmission dynamics. It is an epidemiological feature that is strongly influenced by both pathogen and host traits. At one extreme, limited-term pathogens are characterized by the rapid onset of infection, which then fades away within days to months owing to the host’s efficient immune response or host death. At the other extreme, once an infection occurs, some pathogens are chronically present throughout their host’s life. Perhaps counterintuitively, some limited-term pathogens are pervasive in host populations even though infections can be cleared by their hosts’ immune responses. Such epidemiological dynamics may be a direct result of demographic parameters. For example, limited-term pathogens may become persistent in sink communities that are constantly fed by immigrants from uninfected areas, or in host communities with high birth and mortality rates [5–8]. Alternatively, such epidemiological dynamics may be caused by a waning immune response, in which diminishing immune function with time allows reinfection of individual hosts that were previously immunized (e.g., [9–15]). In other cases, some limited-term pathogens (including influenza, severe acute respiratory syndrome coronavirus 2, *Borrelia*, and *Plasmodium*) are highly prevalent even in hosts with competent and efficient immune responses because the pathogens readily evolve antigenic variation that enables new strains to reinfect hosts with immune defenses that cleared prior infections [16–21]. In many cases, it is not known which of these mechanisms, or possibly others, are responsible for the high prevalence and persistence of limited-term pathogens in host species that can nevertheless clear infections through their immune responses.

*Bartonella* is a genus of intracellular bacteria that primarily infect mammals, including rodents, and can cause disease in some domestic animals and people [22]. Various *Bartonella* species, including *Bartonella grahamii*, *Bartonella krasnovii*, *Bartonella taylorii*, and *Bartonella tribocorum*, have limited-term infection kinetics in their rodent hosts. In those species, bacterial loads in the blood typically rise after 4−10 days post-inoculation (d.p.i.), until they approach a peak at 10–30 d.p.i., and the infection is cleared within 50–80 d.p.i. Even though both cellular and humoral immunity seem to be necessary for the complete eradication of *Bartonella* [23], previous studies agree with the major role of the latter, particularly *Bartonella*-specific immunoglobulin G (IgG) antibodies in murine models [24–27]. For instance, in a *Bartonella birtlesii* infection model, CD4- knockout mice presented higher bacterial titers and longer bacteremia, while CD8- knockout mice presented similar infection dynamics to the wild-type mice [28]. Moreover, experimentally infected cotton rats (*Sigmodon hispidus*) and cats (*Felis catus*) failed to develop infection upon re-exposure to the same strain, indicating that they had protective immunological memory [29, 30].

Rodent–*Bartonella* communities in the sand dunes of Israel’s northwestern Negev Desert provide, for several reasons, an excellent system for exploring the fascinating puzzle of limited-term pathogens that are paradoxically pervasive. First, *Bartonella* infections induce specific IgG antibodies that allow the rodents to clear the infections within ~ 80 d.p.i. [25]. Despite this efficient immunological response, 75–100% of the rodents in this region are infected with *Bartonella* at any given time [31–33]. Second, these are source rodent communities in which the same host individuals have lifetimes of about 1 year and are only negligibly affected by these bacterial infections [25, 32, 34]. Moreover, in most years, the appearance of new juveniles is restricted to about 2 months of the year [32]. Thus, it is unlikely that *Bartonella* persistence is a result of flea transmission between infected rodents and new susceptible individuals entering the population. Third, in this region, *Bartonella* bacteria circulate in host communities composed of three rodent species: *Gerbillus andersoni* (de Winton, 1920), *Gerbillus gerbillus* (Olivier, 1801), and *Gerbillus pyramidum* (Thomas, 1919) (presently, *Gerbillus pyramidum* is known as *Gerbillus floweri*, but we use the original name for consistency between studies). Thus, interactions between *Bartonella* and the host’s immune system may depend on the particular host species, which could have important consequences for the overall epidemiological dynamics in multispecies communities. Fourth, from a practical standpoint, this system allows long-term epidemiological experiments in three wild rodent species studied under seminatural conditions (e.g., using fresh plant matter as a water source and sand as bedding), including the cultivation, inoculation, and quantification of *Bartonella* strains derived from nature, and the simultaneous assessment of the rodents’ immune responses [25, 35]. Finally, understanding the interactions of *Bartonella*—a diverse and globally distributed genus that includes emerging and re-emerging pathogens [36, 37]—and their natural hosts will shed light on the mechanisms responsible for *Bartonella* prevalence in nature.

In this study, we test the hypothesis that at least one of the rodent host species exhibits a waning immune response to *Bartonella*, which allows reinfection. To that end, we inoculated adult captive males of all three rodent species with the same *Bartonella* strain. We quantified and compared their bacterial loads and *Bartonella*-specific IgG antibody titers over 139 days post-inoculation, and then for 60 days following reinoculation with the same strain. Contrary to our hypothesis, however, we found a strong and long-lasting *Bartonella*-specific IgG antibody response with protective immunological memory in all three rodent species, which prevented infection upon re-exposure of individuals to the same *Bartonella* strain. Therefore, other eco-evolutionary factors must explain the persistence of *Bartonella* in these rodent populations in nature.

## Methods

### Experimental approach and design

To start the experiment (day 0), we inoculated five males each of *G. andersoni*, *G. gerbillus*, and *G. pyramidum* with *B. krasnovii*. Prior to starting the experiment, we confirmed that all of the individual rodents were *Bartonella* negative by bleeding them at 1–2 weeks before inoculation and examining their samples molecularly and serologically. Then, starting on the day of the first inoculation, and thenceforth every 10–20 days, we simultaneously sampled blood and plasma from all individuals. To quantify *Bartonella* bacterial loads, we extracted DNA from those samples and performed a real-time quantitative polymerase chain reaction (qPCR). In parallel, we used enzyme-linked immunosorbent assays (ELISA) to quantify the hosts’ *Bartonella*-specific IgG antibody levels. At day 140, after almost all of the infected rodents had become negative for *Bartonella* (except for one *G. pyramidum*), we reinoculated all individuals with the same *Bartonella* strain. We then followed their bacterial loads and *Bartonella*-specific IgG antibody kinetics for an additional 60 days. This timeline was chosen to balance (i) the interval between the two inoculation events, (ii) the number of rodents that cleared their primary infection before reinoculation, (iii) the number of rodents that survived to the end of the experiment, and (iv) to approximate reinfection dynamics that would be relevant in nature. In addition to the 15 experimental animals, we inoculated one control individual of each species at day 0 with phosphate-buffered saline (PBS). Then, on day 140, the three control animals were inoculated with the same *Bartonella* strain, and were sampled along with the 15 experimental rodents during all bleeding events. As an additional control for the reinoculation procedure, we also inoculated a *Bartonella*-negative *G. andersoni* with PBS on day 140 and sampled its blood during all sequential bleeding events.

### Study organisms

The three rodent species in our study coexist in the sand dunes of the northwestern Negev Desert in Israel [34, 38, 39]. All of the individual animals that were used in the experiments were from a laboratory colony maintained by HH. This colony consists of the progeny of wild rodents that were born and have been raised in the laboratory for ~ 6 years. These rodents have never been exposed to ectoparasites or any *Bartonella* species, nor have they received any drug treatment. The individuals used in our study were all non-reproductive adult (1.6–3.4 years old) males, with average body masses of 42.4 ± 1.25, 31.9 ± 1.58 and 74.5 ± 2.21 g, for *G. andersoni*, *G. gerbillus* and *G. pyramidum*, respectively. Nonreproductive adult males rather than adult females of reproductive age were used in this study to avoid the variability that is associated with the menstrual cycle. These males were expected to be representative of the entire population of each species. Throughout the year, males and female hosts show similar *Bartonella* prevalence levels, and only non-reproductive adults are present in natural populations during the autumn, summer, and winter [32]. Animals were kept individually in plastic cages (34 × 24 × 13 = 10,608 cm^3^) on a 1-cm layer of autoclaved sand. The cages were housed in an animal facility at an ambient temperature of 24.5 ± 1 °C and a photoperiod of 12-h:12-h dark:light. The rodents were provided daily with millet seeds *ad libitum* and alfalfa as a water source. The *Bartonella* strain used was *B. krasnovii* A2. This strain was isolated from *G. andersoni* blood, and it belongs to the most common lineage that infects the rodents in this study system [33].

### Inoculation and quantification of bacterial loads

To prepare the *Bartonella* inoculum from the frozen stock of *B. krasnovii* A2 strain, we spread cells to produce confluent lawns on two chocolate agar plates, subsequently collected all the cells from the plates, and diluted the bacteria in 5 ml of PBS to reach a concentration of 3.2 × 10^8^ colony-forming units per milliliter. We chose this concentration because 0.1 ml of the inoculum provides the minimum number of bacteria required for 100% success of inoculations with *B*. *krasnovii* A2 strain, and it also lies well within the range of natural loads of *Gerbillus* rodents [40, 41]. We then intradermally injected 100 μl of the inoculum into each individual rodent, using a 30G needle. A successful inoculation resulted in the formation of a bleb. We chose intradermal injections over subcutaneous, intramuscular, intraperitoneal, intravenous, and intraocular injections as this method best simulates the flea-borne transmission experienced by the pathogen in nature [30, 40, 42]. We performed inoculations under isoflurane anesthesia; immediately afterward, we returned rodents to their cages and confirmed daily thereafter that there were no skin reactions.

We assessed each rodent’s bacterial load by collecting 150–250 μl of blood from the retro-orbital sinus under general isoflurane anesthesia. Once the individual was fully anesthetized, we positioned it in lateral recumbency and applied a drop of local anesthesia (Localin; Fischer Pharmaceutical Labs, Tel Aviv, Israel) to one of its eyes. We collected blood using capillaries coated with 0.14% anticoagulant (ethylenediaminetetraacetic acid) and stored it in ethylenediaminetetraacetic acid blood collection tubes (Microvette, 500 μl; SARSTEDT, Nümbrecht, Germany) at − 80 °C for later molecular analyses. This animal handling protocol was approved by the Committee for the Ethical Care and Use of Animals in Experiments of Ben‐Gurion University of the Negev (permit number IL‐59‐09‐2015). Animal populations originally captured from the wild were held in the Hawlena laboratory with the permission of the Israel Nature and Parks Authority (permit number H1877/2017).

We extracted DNA from 50 μl of each blood sample, using a QIAamp BiOstic Bacteremia DNA Kit (QIAGEN, Hilden, Germany), following the manufacturer’s instructions. We included a negative control in each extraction session, in which all of the reagents were added to sterile PBS instead of to blood. We quantified bacterial loads using gene copy number as a proxy, by qPCR (CFX Connect System; Bio-Rad, Hercules, CA). We targeted the citrate synthase (*gltA*) gene using 2× qPCRBIO Fast Probe Blue Mix Hi-ROX (PCR Biosystems, London, UK), 400 nmol l^−1^ of the *gltA* forward primer 5’-GGATTTGGTCACCGAGTCTATAAA-3’, 400 nmol l^−1^ of the *gltA* reverse primer 5’-AAGAAGCGGATCGTCTTGAATAT-3’, 50 nmol l^−1^ of probe 5’-CCACGTGCAAAAATCATGCAAAAAACCTGTCA-3’ (Primerdesign, Chandlers Ford, UK), and 2 μl of DNA in a total volume of 20 μl. The qPCR conditions were 3 min at 95 °C, followed by 41 cycles of 10 s at 95 °C, and 30 s at 60 °C. To estimate absolute copy numbers, we included in each run a tenfold serial dilution standard curve of *B. krasnovii* A2 strain, which was calibrated using colony-forming unit counts.

### *Bartonella*-specific IgG antibody quantification

After collecting the blood used to quantify the bacterial load, we centrifuged the remaining blood for 10 min at 1000 *g* and 4 °C. We then collected the plasma and stored it at − 80 °C for later immunological analyses. We developed a quantitative ELISA protocol and used it to assess the *Bartonella*-specific IgG antibody kinetics during infection and reinfection of the three host species, as a measure of their humoral immune response against *Bartonella*. We prepared heat-killed *B. krasnovii* A2 strain antigens by heating cells in PBS at 56 °C for 1 h, and we used the antigens to coat multiwell plates for the assays. We determined the *Bartonella*-specific IgG antibody levels in plasma samples by ELISA, following Eidelman et al. [25]. Briefly, we coated each well with 100 μl of *Bartonella* antigens corresponding to a concentration of 1 × 10^6^ cells ml^−1^ in a carbonate-bicarbonate coating buffer, pH 9.6. We put the coated plates in a humidified chamber at 4°C overnight. We blocked plates for 2 h at 37 °C, using 0.5% skim milk in PBS. We then added diluted plasma samples in a blocking solution, and incubated the plates overnight in a humidified chamber at 4 °C. We detected *Bartonella*-specific IgG antibodies using horseradish peroxidase-conjugated rabbit anti-gerbil IgG on a 3,3',5,5'-tetramethylbenzidine substrate. We terminated color development using 3,3',5,5'-tetramethylbenzidine stop solution (SeraCare, Milford, MA), and measured optical absorbance at 450 nm.

For the standard curve, we used *Bartonella*-specific IgG antibodies. Briefly, we purified IgG from serum obtained from *Bartonella* hyperimmune rodents, using protein G spin columns. We then incubated 1 ml of purified IgG overnight with heat-killed *B. krasnovii* A2 strain at 4°C. After centrifugation, we removed the supernatant and washed the pellet five times with wash solution (SeraCare). To elute *Bartonella*-specific IgG antibodies, we resuspended the washed pellet in glycine elution buffer, pH 2.5, followed immediately by the addition of neutralization buffer (Tris HCl 1M, pH 9). We mixed the contents of each tube thoroughly, then centrifuged the tubes for 10 min at 15,000 *g* and 4 °C. We then collected the supernatant and determined the IgG concentration by measuring absorbance at 280 nm in a Nanodrop spectrophotometer, using the automated calculation that is standardized to mammal IgG antibodies. In addition to the plasma samples of the infected rodents, each ELISA plate included all standard dilutions, a blank solution, in which all of the reagents were added to PBS instead of to plasma, and a plasma sample from one of the control rodents.

### Statistical analyses

We performed one-way ANOVA to test for differences in bacterial loads and IgG antibody kinetics between the infected individuals of the three host species (independent variable). The dependent variables were as follows: (i) the peak bacterial load; (ii) the overall bacterial load, which we defined as the area under the bacterial dynamics curve divided by the total number of days; (iii) the rate of antibody increase, defined as the maximum rate of *Bartonella*-specific IgG antibody fold change; (iv) the day post-infection of the peak antibody level; (v) the peak antibody level; and the overall antibody response, defined as the area under the *Bartonella*-specific IgG antibody dynamics curve divided by the total number of days for (vi) the primary and (vii) the secondary immune responses.

To test for an association between bacterial loads and immune kinetics, we performed two analyses of covariance (ANCOVA), with species as the independent variable. One ANCOVA used day post-infection of peak antibody level and peak bacterial load as the dependent variable and covariate, respectively. The other ANCOVA used the overall antibody response and overall bacterial load as the dependent variable and covariate, respectively. In both tests, we also included the interactions between the host species and the covariate. To compare the primary and secondary *Bartonella*-specific IgG antibody responses, we performed repeated measures ANOVA tests, with the day post-infection of peak antibody level, the peak antibody level, and overall antibody response as the dependent variables. For all of the repeated measures ANOVA tests, the immune response stage (primary or secondary) and species were used as the within and between factors, respectively. To standardize these comparisons between the primary and secondary responses, we only used data for the first 60 days of the former. We performed all post hoc comparisons between species using Tukey tests.

All analyses were conducted in R [v4.1.3] [43]. We calculated the area under the dynamics curves using the auc function in the flux package [44]. We calculated the maximum fold change rate of antibody increase using the easylinear function in the growthrates package [45], assuming maximum intervals of two time points.

## Results

### General results

**Fig. 1:**
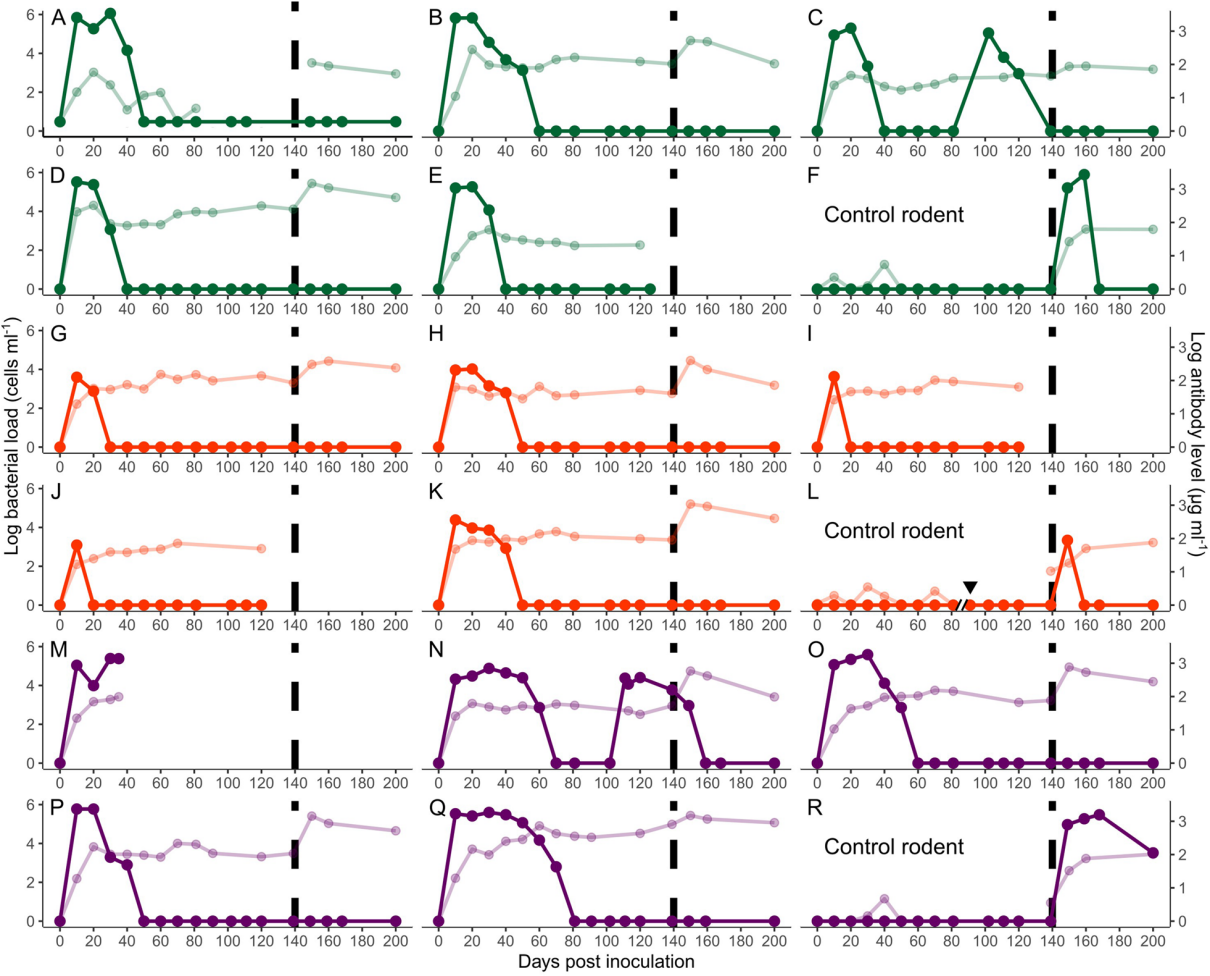
**a**–**r** Bacterial and immune dynamics of individual rodents. Bacterial loads (solid lines, left* y*-axis) and *Bartonella-*specific immunoglobulin G (IgG) antibody levels in plasma (faded lines, right *y*-axis) of five *Gerbillus andersoni* (green; **a**–**f**), five *Gerbillus gerbillus* (red; **g**–**l**), and five *Gerbillus pyramidum* (purple; **m**–**r**) were tracked in a 200-day experiment. All individuals were inoculated with wild-type *Bartonella krasnovii* A2 strain at day 0 and then reinoculated at day 140 (vertical dashed line), except for the three control rodents that were first inoculated with phosphate-buffered saline and only inoculated with *Bartonella* at day 140. All inocula contained 10^7^ colony-forming units. Data are log (*y* + 1) transformed to facilitate comparison between species and parameters. **e**, **i**, **j**, and **m** Bacterial and immune dynamics are truncated due to the unexpected death of the individuals before the end of the experiment. **l** The axis break and inverted triangle indicate the switch between control individuals due to the death of the original rodent

We inoculated five males each of *G. andersoni*, *G. gerbillus*, and *G. pyramidum* with the same strain, *B. krasnovii* A2. We followed the bacterial dynamics and *Bartonella*-specific IgG antibody kinetics for 139 days after the primary inoculation, and then for an additional 60 days following reinoculation with the same strain. In parallel, we sampled the blood of three control rodents (one of each species) that were first inoculated with PBS and only inoculated with *Bartonella* at day 140 (Fig. 1). Of the 18 individual rodents, 13 remained healthy, maintaining a stable body mass throughout the 200 days of the experiment. These were included in all of our analyses. The other five rodents were either excluded from all of the analyses or included in only some of them. These five included one *G. pyramidum* that was euthanized due to poor physical condition at day 40 (Fig. 1m). It was excluded from all analyses. Three other individuals either did not recover from the anesthesia at day 125 of the experiment (one *G. gerbillus*; Fig, 1i) or were found to have died, for no visible reason, on days 125 and 120 of the experiment [one *G. andersoni* (Fig. 1e) and one *G. gerbillus* (Fig. 1j)]. They were excluded from all analyses related to the secondary immunological response to reinoculation. Moreover, in the analysis of the overall antibody response to the primary inoculation, we assigned at day 139 the species-specific mean values for the three aforementioned rodents that did not survive to that day. The fifth individual was a control *G. gerbillus*, which died for no visible reason at day 80. It was replaced by another *Bartonella*-negative *G. gerbillus* individual. Accordingly, Fig. 1l shows the combined dynamics of these two individuals.

Blood was successfully sampled and processed for all surviving animals at all the planned sampling dates [i.e., days 0, 10, 20, 30, 40, 50, 60, 70, 81, 102 (only for bacterial load), 111 (only for bacterial load), 120, 139, 149, 159, 169 (only for bacterial load), and 200 d.p.i.]. In addition, four individuals had a questionable infection status at day 81 (i.e., amplification was successful only at very late quantification cycles, Cq > 39), so we bled them again at day 91 to confirm their infection status. At day 113, we also quantified the bacterial load of the single *G. pyramidum* individual that showed evidence of recurrent bacteremia at day 111 (Fig. 1n), which confirmed that it was *Bartonella*-positive. The only surviving participant that was not bled as planned (at days 120 and 139) was a *G. andersoni*, which was *Bartonella*-negative between days 50 and 102 (Fig. 1a). Due to its poor condition, we decided to reduce the number of bleeding events for this animal to allow it to recover some body mass. Thus, in the analysis of the overall antibody response to the primary inoculation, we assigned the *G. andersoni*-specific mean value to this individual on day 139.

### Bacterial and immune dynamics

The bacterial load and antibody quantifications were *Bartonella*-specific, as the control rodents remained *Bartonella*-negative throughout the first experimental stage (days 0–140), with no noteworthy rise in their antibody levels, whereas they developed an infection and elevated antibodies upon inoculation during the second experimental stage (days 150–200; Fig. 1, control rodents). In contrast, all the rodents that were inoculated with *Bartonella* became infected and developed antibodies during the first stage (Fig. 1). The peak bacterial loads and the overall bacterial loads were significantly different between the host species (Table 1), with *G. gerbillus* exhibiting lower loads than the other two species (Tukey post hoc tests, peak load, *P* = 0.017 for the comparison with *G. andersoni*, *P* = 0.019 for the comparison with *G. pyramidum*; overall load, *P* = 0.0338 for the comparison with *G. andersoni*, *P* = 0.00961 for the comparison with *G. pyramidum*; Fig. 1), which had similar bacterial loads (Tukey post hoc tests, peak load, *P* = 0.994; overall load, *P* = 0.659; Fig. 1).

**Table 1:** Comparisons between the infection and immune dynamics of the three rodent species

Parameter/associations	*Gerbillus andersoni*	*Gerbillus gerbillus*	*Gerbillus pyramidum*	*F*_*df*_, *P*^a^
Peak bacterial load (cells ml^−1^)	351,600 ± 87,479	8804 ± 4115	363,175 ± 106,916	*F*_2,11_ = 7.37, *P* = 0.009**
Overall bacterial load (cells ml^−1^)	49,389 ± 12,860	1057 ± 547	64,906 ± 18,656	*F*_2,11_ = 7.59, *P* = 0.00848**
Day post-infection of peak antibody level	42.0 ± 19.6	66.0 ± 2.45	74.8 ± 24.4	*F*_2,11_ = 0.981, *P* = 0.406
Rate of antibody increase during primary infection (μg ml^−1^ day^−1^)	0.320 ± 0.0575	0.344 ± 0.0266	0.290 ± 0.0187	*F*_2,11_ = 0.422, *P* = 0.666
Rate of antibody increase during secondary infection (μg ml^−1^ day^−1^)	0.123 ± 0.0275	0.180 ± 0.0300	0.172 ± 0.0403	*F*_2,7_ = 0.663, *P* = 0.545
Peak antibody levels (μg ml^−1^)	149 ± 63.5	110 ± 20.0	309 ± 169	*F*_2,11_ = 1.22, *P* = 0.332
Overall primary antibody response (μg ml^−1^)	82.8 ± 31.5	67.8 ± 11.3	145 ± 67.6	*F*_2,11_ = 1.04, *P* = 0.386
Overall secondary antibody response (μg ml^−1^)	287 ± 169	339 ± 139	549 ± 146	*F*_2,8_ = 0.842, *P* = 0.466
Day post-infection of peak antibody level (days) versus peak bacterial load (cells ml^−1^)	-1.11 × 10^–4^* x* + 81.1, *R*^2^ = 0.247	9.36 × 10^–5^* x* + 65.2, *R*^2^ = 0.0250	1.22 × 10^–4^* x* + 30.5, *R*^2^ = 0.284	*F*_2,8_ = 1.43, *P* = 0.295
Overall primary antibody response (μg ml^−1^) versus overall bacterial load (cells ml^−1^)	8.49 × 10^–4^* x* + 40.9, *R*^2^ = 0.120	8.77 × 10^–3^* x* + 58.6, *R*^2^ = 0.180	2.94 × 10^–3^* x* – 45.5, *R*^*2*^ = 0.659	*F*_2,8_ = 0.872, *P* = 0.454
Difference in the day post-infection of peak antibody level of the primary and secondary responses	7.50 ± 7.38	46.7 ± 8.52	30.0 ± 7.38	*F*_2,8_ = 6.23, *P* = 0.023
Difference in the peak antibody level (μg ml^−1^) of the secondary and primary immune responses	369 ± 215	503 ± 248	801 ± 215	*F*_2,8_ = 1.05, *P* = 0.394
Difference in the overall antibody response (μg ml^−1^) of the secondary and primary immune responses	219 ± 124	281 ± 144	454 ± 124	*F*_2,8_ = 0.948, *P* = 0.427

Means ± SE of the infection parameters, the immunoglobulin G (*IgG*) antibody kinetics parameters, and the correlations between parameters. The correlations were conducted between immune and bacterial parameters and between the primary and secondary IgG antibody kinetics parameters that were quantified in *G. andersoni*, *G. gerbillus*, and *G. pyramidum* after the initial inoculations (primary response) and reinoculations (secondary response) with* Bartonella*

*Asterisks* indicate statistically significant differences (** P < 0.01)

^a^Statistical results for either the species effects (for specific parameters) or the interactions between the species effect and the independent variable in question (for correlations between parameters) (the definitions of the parameters are provided in the statistical analyses section)

**Fig. 2:**
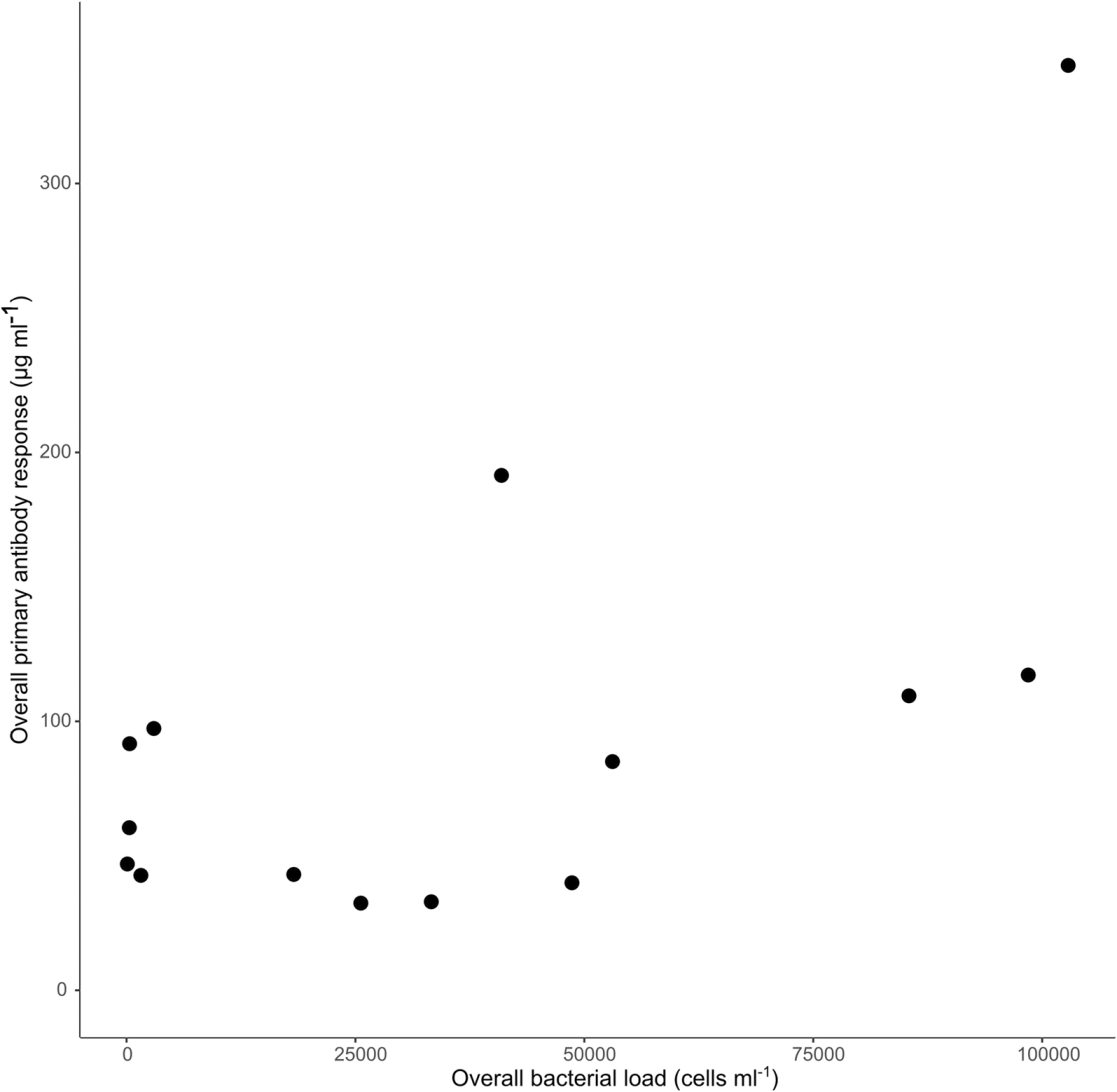
Primary immune response strength. Correlation (*r* = 0.49) between the overall primary *Bartonella*-specific IgG antibody response (the area under the antibody dynamics curve divided by the total number of days) and the overall bacterial load (the area under the bacterial dynamics curve divided by the total number of days). Each point represents an individual rodent

**Fig. 3:**
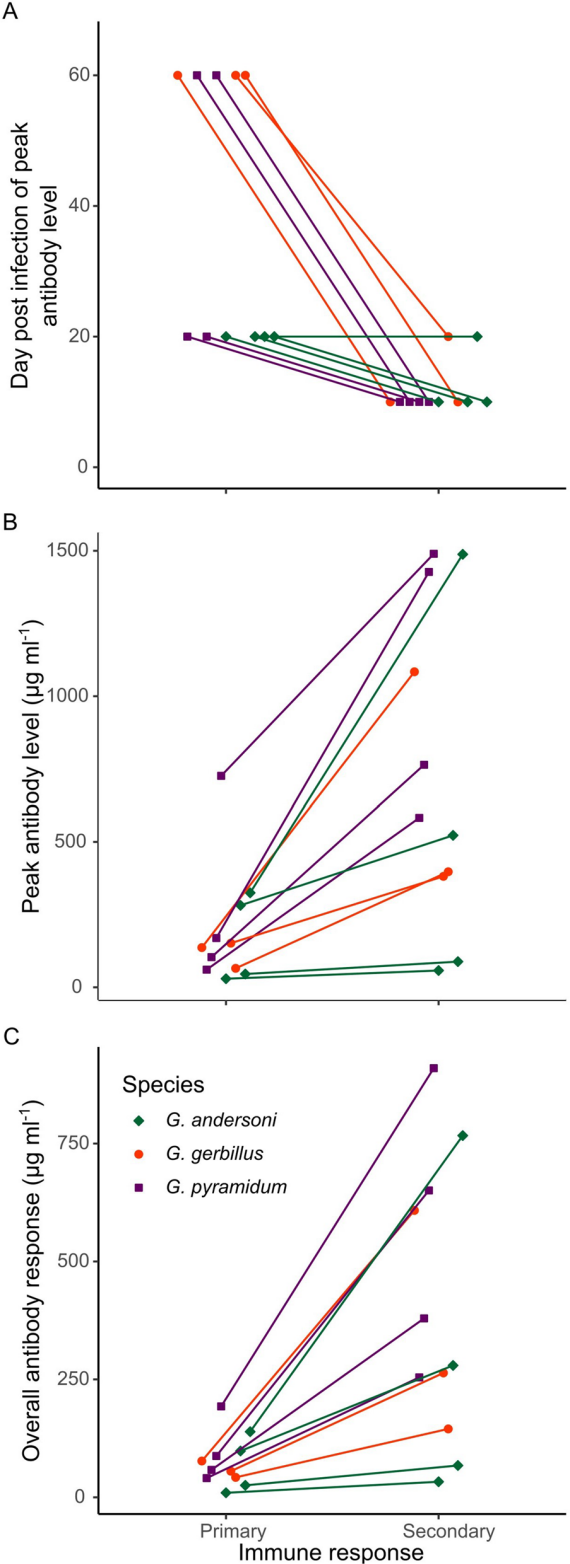
**a**–**c** Primary and secondary immune responses. Differences between the primary and secondary immune responses of *Gerbillus andersoni* (green), *Gerbillus gerbillus* (red), and *Gerbillus pyramidum* (purple), which are quantified by** a** the day post-infection of peak *Bartonella*-specific IgG antibody levels (determined based on the respective day of inoculation), **b** peak antibody level, and **c** overall antibody response (the area under the antibody dynamics curve divided by the total number of days). To standardize the comparisons between the primary and secondary responses, we used only data for the first 60 days of the former. Lines connect measures of the same individual rodent. Points and lines are horizontally jittered to make overlapping points visible

The day post-infection of peak antibody level was not significantly correlated with peak bacterial load (*F*_1,10_ = 0.0002, *P* = 0.989). However, the overall antibody response was positively correlated with overall bacterial loads (*F*_1,10_ = 6.56, *P* = 0.0283; Fig. 2). All infected rodents cleared their initial infection after 10–70 days (Fig. 1). However, one individual *G. andersoni* (Fig. 1c) and one individual *G. pyramidum* (Fig. 1n) experienced recurrent infections, with the *G. pyramidum* experiencing a recurrent infection prior to, but close to, the second inoculation (days 111–149 d.p.i.). All previously infected rodents mounted a secondary immune response following the repeated inoculation, as expected for animals with immune memory. Moreover, as expected, the humoral response that ensued was more rapid (*F*_1,8_ = 35.1, *P* = 0.000351; Fig. 3a) and antibody levels were higher in the secondary response than in the primary response [*F*_1,10_ = 18.6, *P* = 0.00152 for peak antibody level (Fig. 3b), and *F*_1,10_ = 18.6, *P* = 0.00155 for overall antibody response (Fig. 3c)]. Importantly, it appeared that the primary immune response after the first inoculation protected all rodents, as none of the rodents developed bacteremia following reinoculation (Fig. 1).

**Fig. 4:**
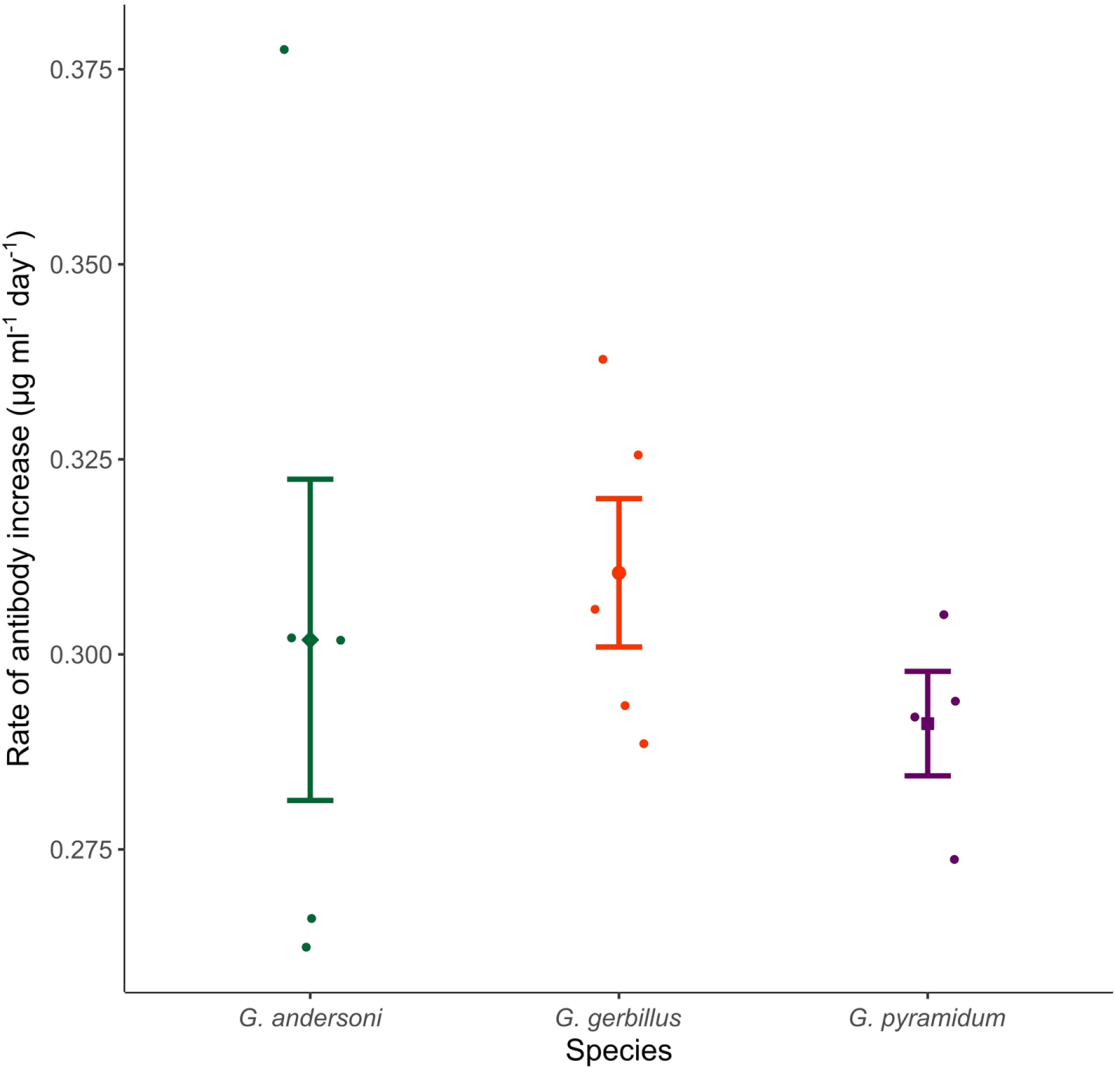
Species-specific rate of antibody increase. Means ± SE of the maximum *Bartonella*-specific IgG rate of antibody increase in *Gerbillus andersoni* (green), *Gerbillus gerbillus* (red), and *Gerbillus pyramidum* (purple) after the initial inoculations. Raw data points are horizontally jittered to make overlapping points visible

Most features of the IgG antibody kinetics, in addition to the associations between IgG kinetics and bacterial dynamics and between the primary and secondary IgG antibody kinetics, were not significantly different between the three species (Table 1). The only association that differed significantly among species was between the days post-infection when peak antibody levels were observed in the primary and secondary responses, with *G. gerbillus* having the greatest shortening of the immune response time to the secondary inoculation compared to the primary inoculation (Tukey test, *P* = 0.0204 for the *G. gerbillus*-*G. andersoni* comparison; *P* = 0.350 for the *G. gerbillus*-*G. pyramidum* comparison; *P* = 0.139 for the *G. andersoni*-*G. pyramidum* comparison; Table 1; Fig. 3a). There was also a tendency for a higher rate of antibody increase in *G. gerbillus* than in *G. pyramidum* during the primary infection (Fig. 4), but this difference was not statistically significant (a split comparison between *G. gerbillus* and *G. pyramidum*, *F*_1,7_ = 2.48, *P* = 0.160; Table 1).

## Discussion

We profiled the dynamics of a natural *Bartonella* isolate as it infected captive individuals of three rodent species that coexist in nature. We also tracked *Bartonella*-specific IgG antibody levels in these animals over 140 days after the first inoculation and 60 additional days following their re-exposure to the same strain. In nature, the mean longevity of the three rodent species ranges from 6.5 to 12 months [34, 46, 47]. Thus, the 200-day duration of this experiment likely allowed for a decent approximation of the infection dynamics that these rodents may experience in nature. Considering the high prevalence of *Bartonella* in these species of rodents in the Negev sand dunes, we tested the hypothesis that at least one of them exhibits a waning immune response, which could allow the pathogen to reinfect individuals that cleared prior infections. However, contrary to our hypothesis, we found a strong and long-lasting *Bartonella*-specific IgG antibody response, with a protective immunological memory in all the rodent species, which prevented infection upon re-exposure to the same *Bartonella* strain. In addition, two host individuals showed recurrent bacteremia during the first infection stage. Below, we discuss our findings of a comprehensive immune response, recurrent bacteremia, and species-specific differences in a broader disease ecology context and discuss future avenues of research for investigating the puzzle of limited-term *Bartonella* infections that are nonetheless pervasive. Altogether, insights from this study constitute an initial step toward a better understanding of the interplay between pathogen and host traits, and how the interplay of those traits influences epidemiological dynamics.

### A comprehensive immune response against *Bartonella*

Longitudinal studies have shown that *Bartonella* species may be highly prevalent, and that the same strains can be repeatedly detected even after a nonbacteremic period [48–51], leading to the hypothesis that the host immune response against these species wanes [48, 52]. Contrary to this hypothesis, our results provide several lines of evidence suggesting that all three of the tested rodent species responded to the inoculation by mounting a strong, efficient, and long-lasting antibody response, which conferred protection and prevented bacteremia following reinoculation at day 140 d.p.i. First, all of the individuals that were included in the analyses mounted *Bartonella*-specific antibody responses within 10 days of the initial inoculation. Antibody levels then increased and approached local peak levels, which were maintained at relatively high levels or even increased during the remaining period until the repeat inoculation was performed. Importantly, *Bartonella*-specific antibody levels remained high long after the rodents managed to clear the infections. Second, the magnitude of the specific antibody response was positively correlated with the bacteremia load. Third, the specific antibody response increased in all rodents and was more rapid upon reinfection, suggesting immune memory and improved IgG antibody response upon re-exposure to the bacteria. Finally, none of the rodents that were reinoculated developed bacteremia or showed recurrent bacteremia, and we found evidence that *Bartonella*-specific IgG antibodies synthesized upon first inoculation efficiently cleared reinoculated *Bartonella* even in the individuals displaying recurrent bacteremia (see “Recurrent bacteremia” section). The absence of bacteremia did not appear to be a result of a low-quality or non-viable inoculum, as the second inoculum was prepared from the same *Bartonella* isolate, using the same procedure as for the first inoculum, and had a bacterial concentration that was similar to that of the first inoculum. Moreover, the bacterial dynamics displayed in the three control rodents, which were inoculated with the second inoculum, were similar to those of the rodents infected with the first inoculum. Thus, our results suggest that the observed specific antibody response most likely prevented *Bartonella* re-establishment in the rodents upon reinoculation.

These findings of the limited-term nature of infections with *Bartonella krasnovii* A2 strain, likely owing to the long-lasting *Bartonella*-specific IgG antibody response of its rodent hosts, align with observations of other *Bartonella* species (e.g., *Bartonella grahamii*, *Bartonella taylorii*, and *Bartonella henselae*) in a variety of reservoir hosts, including house mouse (*Mus musculus*), cotton rats (*S. hispidus*), and cats (*F. catus*), which illustrated similar in vivo bacterial dynamics, antibody kinetics, antibody-mediated clearance of bacteremia, and failures of reinfection [24, 27, 29, 30]. Our findings also add to experimental evidence showing that IgG antibodies activate the complement system and inhibit *Bartonella* adhesion to erythrocytes (reviewed in [53]). Taken together, this evidence suggests that phylogenetically distant reservoir hosts have similar strategies for clearing *Bartonella* infections. These strategies are based on the high turnover rate of erythrocytes and the development of IgG antibodies that prevent bacterial binding to host erythrocytes when *Bartonella* are periodically seeded from other niches (see below; [53]). Thus, the results of our experiment broaden the universal view of the interactions between *Bartonella* and their reservoir hosts and suggest that if *Bartonella* did not continue to evolve rapidly, they would likely be eliminated from natural communities.

### Recurrent bacteremia

We observed recurrent bacteremia in one *G. andersoni* host and one *G. pyramidum* host. In these individuals, *Bartonella* cells reappeared in the bloodstream within 30–40 days of their disappearance despite the fact that both individuals mounted strong *Bartonella*-specific responses upon inoculation. There are several possible explanations for this pattern of recurrence. First, it is possible that the infection had never been cleared from the blood of these two rodents, but that its level decreased below detectable levels [48]. However, as infections in the other inoculated rodents in the current study, as well as in 20 *G. andersoni* that were inoculated with the same *Bartonella* strain in a previous study [25], never lasted for more than 70 days, this explanation seems implausible. Second, the recurrent bacteremia may have been a result of a waning immune response [52]; however, this is unlikely, as in addition to displaying comparable antibody levels to the other rodents, the two rodents in question did not develop secondary bacteremia upon reinoculation (once they had cleared the recurrent infections). 

A third possible explanation is that, upon first inoculation of these two rodent individuals, some bacterial cells remained in—as yet unidentified—cellular niches in host tissues, where they persisted and replicated. Only later, after the rest of the bacteria were cleared, did these latent bacteria re-enter and recolonize the blood stream. The first recognized so-called primary niche in *Bartonella* species was endothelial cells, but additional cellular niches with similar roles were later proposed, including the dermis, lymph nodes, bone marrow, liver, spleen, and the kidney (reviewed in [54]). This hiding–seeding mechanism was proposed as an explanation for the 3- to 6-day interval of recurrent bacteremia that was detected in rodent models and in people infected with *Bartonella quintana* [26, 27, 53]. This mechanism, which allows subpopulations of bacteria to hide and reappear in different niches, could also contribute to the long duration of the IgG antibody response that was observed in our study. However, the “hidden niche” hypothesis alone cannot explain the longer intervals of recurrent bacteremia that were observed in the current study, i.e., 30–60 days of initial bacteremia, followed by 30–40 days of *Bartonella*-negative blood, and then 40–60 days of recurrent bacteremia (Fig. 1c, n). After such long infection intervals, we would expect that, upon their release from the hidden niche, these bacterial cells, which are similar to the cells in the repeated inoculation, would be revealed and immediately targeted by the host immune response. Alternatively, we propose that, in the hidden niche, some *Bartonella* organisms have evolved to escape the specific IgG antibodies (see the below section on antigen escape). This hiding–mutating–seeding scenario that is supported by both the observed bacterial and IgG antibody dynamics, and aligns with immunological evidence from other *Bartonella* species [53], may also be responsible for the long intervals between recurrent bacteremia that were observed in experimental infections of cats [55] and longitudinal field studies (e.g., [48, 50]). This hypothesis should be confirmed by comparing the genome sequences of *Bartonella* in the host’s blood during the peaks of initial and recurrent infections, and testing the cross-reactivity of the IgG response to these two bacterial sources.

Future studies should assess how common recurrent bacteremia is under natural conditions. In natural populations of cotton rats (*S. hispidus*) and deer mice (*Peromyscus maniculatus*), 8–15% of the hosts showed recurrent *Bartonella* infections [48, 50], similar rates to those observed in the current experiment (14%). However, since there is experimental evidence that recurrent bacteremia might be associated with intradermal inoculations, which resemble the vector-borne transmission route (current study; [28]), it is possible that, in the northwestern Negev Desert, flea transmission will even further amplify this phenomenon. Apart from assessing the commonality of this phenomenon, it is important to reveal the exact mechanism underlying recurrent bacteremia for a better understanding of host–pathogen interactions. The challenge of future longitudinal studies in animals and people will be to develop molecular techniques that differentiate between recurrent bacteremia and reinfection by the same strain. The distinction between these two processes, which was enabled here by our experimental set-up, is crucial for understanding pathogen population and community dynamics and for informing effective medical solutions against persistent infections [56].

### Species-specific differences in antibody kinetics

Contrary to our hypothesis that at least one of the rodent species would exhibit a waning immune response, as mentioned above, we found that infection with *B. krasnovii* A2 strain elicited an efficient and protective immune response in all of the species tested. Yet, despite the similar microhabitats of *G. gerbillus* and *G. pyramidum* [34], and the similar body size of *G. gerbillus* and *G. andersoni* [57], our results indicate that the immune response of *G. gerbillus* may be more reactive than those of the two other species. This was demonstrated by the magnitude of the increases in antibody levels following reinfections compared to the primary responses, which were highest in *G. gerbillus* (Fig. 3a). In addition, the mean rate of antibody increase was greater (although not significantly different) in *G. gerbillus* than in *G. pyramidum* (Fig. 4). Finally, both the peak and overall bacterial loads of *G. gerbillus* individuals were significantly lower than those of the two other species. In accordance with these results, *Bartonella* prevalence was lower in *G. gerbillus* populations in the study region compared to populations of the other two species (see supporting data in [31]).

Higher resistance is often observed when hosts are locally adapted to their pathogens [58–62]. Thus, the greater resistance of *G. gerbillus* may indicate that this host is more adapted to *Bartonella* than the two other species. However, considering the sporadic temporal and spatial occurrence of *G. gerbillus* as compared to the steady occurrence of *G. andersoni* and *G. pyramidum* rodents in the natural environment [34], it is unlikely that *G. gerbillus* faces strong selection due to infections with *B. krasnovii* A2 strain. Instead, assuming that *Bartonella* is ahead of its hosts in the evolutionary arms race, the higher bacterial loads in *G. andersoni* and *G. pyramidum* compared to *G. gerbillus* may indicate that *B. krasnovii* A2 strain is more adapted to these more reliable hosts, and can therefore better hide and/or escape from their IgG antibodies [63, 64]. To test this hypothesis, future experiments should compare the bacterial dynamics and immune kinetics of different *Bartonella* strains that are either locally adapted or not adapted to each of these rodent species. The aim of these studies would be to elucidate the missing links between the long-term infection dynamics, immune kinetics, and history of coevolution between these pathogens and their hosts, which is crucial information for understanding patterns of epidemiological dynamics in natural communities.

### Future directions to solve the *Bartonella* pervasiveness puzzle

While the unique strategy by which *Bartonella* persists for weeks within the protected niche of host erythrocytes is consistent with their high prevalence in reservoir hosts, our finding that there is a comprehensive host serological immune response with an efficient memory leaves the puzzle of *Bartonella*’s pervasiveness unresolved. Future laboratory experiments with food-deprived rodents, juvenile rodents, and reproductive female rodents under predation risk should be conducted to confirm our results in communities that better represent the states of these rodent populations in nature. These experiments could address whether a comprehensive immune response against *Bartonella* would also develop under more challenging conditions for hosts than the seminatural conditions provided in the current experiment. In parallel, it is important to follow the changes in infection status of, and *Bartonella* strain composition within, the same rodent individuals over monthly intervals in the field.

Considering that the studies proposed above may provide further support for the existence of a comprehensive immune response against *Bartonella*, we also suggest that other studies should be undertaken that focus on an alternative explanation for the puzzle of *Bartonella*’s pervasiveness, namely, the existence of genetic mechanisms that allow these pathogens to rapidly evade the well-adapted immune responses of their natural hosts. This alternative explanation is in line with the results of a longitudinal study of the dynamics of *Bartonella* observed in a natural population of cotton rats (*S. hispidus*), in which infections of the same individual hosts by *Bartonella* variants from different genogroups often followed one another [65, 66].

Antigenic variation—when pathogen populations evolve to alter surface features targeted by the host immune system—is one of the most widely used escape strategies that allows pathogens to reinfect hosts that have developed an immune response against the original strain (preceding antigenic changes; [67]). In *Bartonella*, at least three genetic mechanisms operate that could lead to rapid antigenic variation. First, contingency loci, which are hypermutable sites on specific genes, may undergo mutations that add or remove repeat units at high rates due to strand slippage during DNA replication. Although they have never been fully profiled in *Bartonella*, an elevated number of mononucleotide repeats in this genus, relative to other bacteria, has been noted [68]. Second, genome comparisons of virulence gene arrays in *Bartonella* revealed high rates of intragenomic recombination events that copy, delete, and hybridize the main versions of these genes with other nearby copies [69–72]. Finally, *Bartonella* share a domesticated prophage that acts as a gene transfer agent, packaging their DNA for transduction. Gene transfer agent-mediated recombination may accelerate antigenic variation, and virulence factor evolution through the exchange of DNA between co-infecting strains in the flea gut or host tissues [73]. Understanding what roles these and other genetic mechanisms for rapid evolution play in the spread and persistence of *Bartonella* may shed light on a universal mystery—the pervasive nature of limited-term pathogens despite efficient host immune responses.

## Conclusions

This study constitutes an initial step toward understanding how the interplay between traits of *Bartonella* and their hosts influences the epidemiological dynamics of these limited-term pathogens in nature.

## Data Availability

The dataset supporting the conclusions of this article is available in the Dryad Digital repository at https://datadryad.org/stash/share/Xi0f1sWaXzrAGMUjaMPMB_uP_xN8OsM_qICsJSpeUiQ, Rodríguez-Pastor et al. 2023.
